# Male courtship preferences demonstrate discrimination against allopatric colour morphs in a cichlid fish

**DOI:** 10.1111/jeb.12074

**Published:** 2013-01-03

**Authors:** P Zoppoth, S Koblmüller, K M Sefc

**Affiliations:** Department of Zoology, University of GrazGraz, Austria

**Keywords:** allopatric divergence, Cichlidae, Lake Tanganyika, male preference, mate choice, reproductive isolation, Tropheus

## Abstract

Whether premating isolation is achieved by male-specific, female-specific or sex-independent assortative preferences often depends on the underlying evolutionary processes. Here we test mate preferences of males presented with females of different allopatric colour variants of the cichlid fish *Tropheus* sp., a Lake Tanganyika endemic with rich geographical colour pattern variation, in which the strength of sexual isolation varies between populations. We conducted two-way mate choice experiments to compare behaviour of males of a red-bodied morph (population Moliro) towards females from their own population with behaviour towards females from four allopatric populations at different stages of phylogenetic and phenotypic divergence. Males courted same-population females significantly more intensely than females of other populations, and reduced their heteromorphic courtship efforts both with increasing genetic and increasing phenotypic distinctness of the females. In particular, females of a closely related red-bodied population received significantly more courtship than either genetically distinct, similarly coloured females (‘Kirschfleck’ morph) or genetically related, differently coloured females (‘yellow-blotch’ morph), both of which were courted similarly. Genetically and phenotypically distinct females (*Tropheus polli*) were not courted at all. Consistent with previous female-choice experiments, female courtship activity also decreased with increasing genetic distance from the males’ population. Given successful experimental and natural introgression between colour morphs and the pervasive allopatry of related variants, we consider it unlikely that assortative preferences of both sexes were driven by direct selection during periods of secondary contact or, in turn, drove colour pattern differentiation in allopatry. Rather, we suggest that sexual isolation evolved as by-product of allopatric divergence.

## Introduction

Assortative and conspecific mate preferences play an important role in maintaining reproductive isolation between species or among morphs within a species (Ritchie, 2007). It has been argued that the pressure to avoid hybridization should be stronger on the sex paying the higher costs for mating mistakes, which is frequently assumed to be the female (Parker & Partridge, 1998; Wirtz, 1999; but see Servedio, 2007). Several empirical examples of female biases in the strengths of assortative preferences support this hypothesis (Saetre *et al*., 1997; Russell *et al*., 2006; Hochkirch *et al*., 2007; Kozak & Boughman, 2009; Verzijden *et al*., 2009; O'Rourke & Mendelson, 2010). Nonetheless, a fairly large proportion of studies comparing male and female mate preferences between sympatric species and in hybrid zones found that male mate choice contributes equally or even more than female choice to sexual isolation (Knight & Turner, 1999; Seehausen *et al*., 1999; Jiggins *et al*., 2001; Shine *et al*., 2002, 2004; Peterson *et al*., 2005; Svensson *et al*., 2007; Pryke & Griffith, 2007; Pierotti *et al*., 2008; Espinedo *et al*., 2010), either because males incur high costs by courtship and by mating mistakes (Albert & Schluter, 2004; Peterson *et al*., 2005; Svensson *et al*., 2007) or because of processes other than reinforcement and direct selection on mating preferences (Jiggins *et al*., 2004; Nosil *et al*., 2007; Pierotti *et al*., 2008; Espinedo *et al*., 2010; Mendelson & Shaw, 2012). For example, sexual isolation can arise as a by-product of allopatric divergence (Coyne & Orr, 2004), in which case a sex bias in the strength of assortative preferences is not necessarily expected. Accordingly, assortative preferences were strong in both males and females of a Lake Malawi cichlid fish species when given the choice between individuals from their own and from an allopatric population (Pauers *et al*., 2010).

The endemic Lake Tanganyika genus *Tropheus* comprises another cichlid species complex with rich allopatric colour pattern variation (Konings, 1998; Schupke, 2003). Most *Tropheus* populations are sexually monomorphic or display only subtle sexual differences in size and colour pattern, and males and females each defend their individual territories. Both sexes employ colour signals to communicate in social and sexual interactions (Nelissen, 1976). For mating, females give up their territories and move into a chosen male's territory for several days to weeks until spawning, upon which they leave the male and mouthbrood their offspring alone for about 4 weeks (Yanagisawa & Nishida, 1991). Genetic analyses indicated that broods are fertilized by a single male each, that is presumably by the females’ temporary social mates (Egger *et al*., 2006). Dispersal of the stenotopic rock-dwellers is restricted by habitat barriers, and significant genetic differentiation as well as slight colour pattern differentiation exists among many adjacent populations, whereas large and longstanding barriers such as river estuaries often separate highly distinct colour variants (Egger *et al*., 2007; Koblmüller *et al*., 2011). The classification of the geographically separated, phenotypically diverse but closely related colour variants into the nominal species is not completely resolved, and populations are typically specified by cheironyms indicating their geographical origin or colour pattern. The most recent common ancestor of the species complex (the genus *Tropheus* excepting the clearly divergent *Tropheus duboisi*) dates back to approximately 1.5 million years ago (Ma; Koblmüller *et al*., 2010). Divergence times between individual populations range between this onset of diversification and the population fragmentation associated with the latest major lake level rise between 10 000 and 20 000 years ago (Egger *et al*., 2007; Koblmüller *et al*., 2011), providing opportunities to examine reproductive isolation at different levels of phenotypic and genetic distance.

Studies of mate choice in laboratory experiments and in an artificially admixed field population suggested that reproductive isolation among allopatric morphs depends (with exceptions) on the extent of phenotypic and genetic differentiation (Salzburger *et al*., 2006; Egger *et al*., 2008, 2010, 2012). Where present, assortative preferences may either have evolved in allopatry or may have been promoted during periods of secondary contact with other morphs precipitated by lake level fluctuations (Sturmbauer *et al*., 2005; Egger *et al*., 2007). With uniparental maternal brood care and a mating system leading to a male-biased operational sex ratio (Sefc, 2008), avoidance of potentially disadvantageous inter-morph matings would be expected to evolve primarily in females. However, inter-morph fertility in experimental crosses (K.M. Sefc, personal observation) as well as natural introgression between morphs and the putative hybrid origin of some colour morphs (Egger *et al*., 2007, 2012) cast doubt on whether heteromorphic matings necessarily incur severe costs on either of the sexes.

In the previous mate choice experiments, females were offered a two-way choice between a homomorphic and a heteromorphic male, and interactions of females with the males were scored in categories representing aggression and mate preference. Although designed as female choice experiments, both male and female preferences could have contributed to the observed patterns, for instance because the intensity of male courtship apparently influences female preferences in within-population interactions (Steinwender *et al*., 2012). In this study, we quantified behaviour in homomorphic and heteromorphic intersexual interactions to examine whether males discriminate against heteromorphic females and modify their behaviour according to their phenotypic and phylogenetic distance from the heteromorphic stimulus females. A two-way male choice design was selected to assess male mate preferences in situations when both homomorphic and heteromorphic choices are available. Behaviours of females, for which the experiment presented a no-choice situation, were scored to assess congruency with male behaviour.

**Fig. 1:**
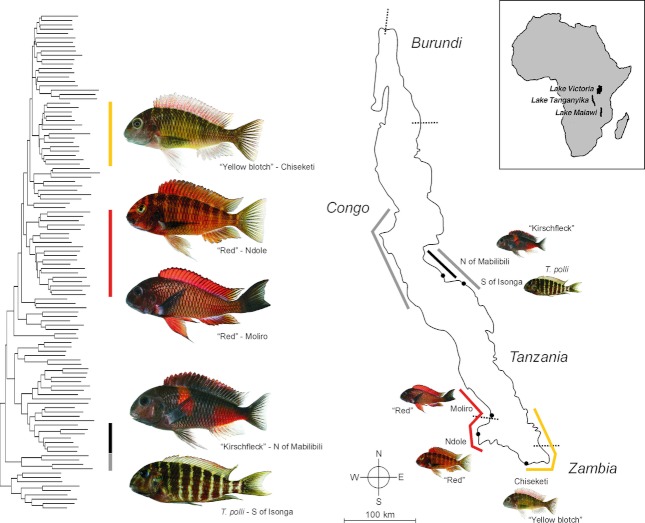
Phylogenetic relationships, colour pattern variation and geographical distribution of the *Tropheus* populations used in this study. The phylogenetic tree of *Tropheus* populations is based on AFLP data and analyses by Egger *et al*. (2007). The coloured bars next to the tree indicate the clades in which the tested populations are placed. Coloured lines along the lake shore represent the distribution of the *Tropheus* morphs (grey lines: striped *Tropheus polli*; black lines: ‘Kirschfleck’ morph; yellow lines: the yellow-blotch morph; red lines: the red-bodied morph). Photographs courtesy of Wolfgang Gessl (www.pisces.at).

The tested males belong to a red-bodied morph occurring in southern Lake Tanganyika (population Moliro, Fig. 1), proposed alternatively to represent its own species *Tropheus* sp. ‘red’ (Konings, 1998) or belong to *Tropheus moorii* (Egger *et al*., 2007). Females of the red-bodied morph displayed strictly assortative mating preferences vis-à-vis several distinct morphs in previous studies (Egger *et al*., 2008; Hermann, 2011; H. Brindl & K. M. Sefc unpublished), but did not discriminate against a closely related and similarly coloured population (Egger *et al*., 2010). For this study, the heteromorphic stimulus females were chosen from populations with different degrees of genetic and colour pattern differentiation from the Moliro population, namely a genetically closely related and similarly coloured population of the same red-bodied lineage (Ndole), a related population with a distinct yellow-blotch colouration (population Chiseketi, *T. moorii*), a genetically distinct population with a red ‘Kirschfleck’ (cherry-blotch) colour pattern (assigned to *Tropheus* sp. ‘black’ by Konings, 1998) and a genetically and phenotypically distinct population assigned to *Tropheus polli* (Fig. 1).

## Materials and methods

The fish were wild caught adults imported by an ornamental fish trader. Prior to the experiments, the fish were weighed, measured and transferred into individual aquaria (60 × 30 × 30 cm) equipped with internal box filters, heaters and hollow bricks and illuminated with overhead white light in a 12:12 h light:dark cycle.

Experimental setup was analogous to the female-choice design developed for *Tropheus* by Egger *et al*. (2008, 2010). Experimental tanks (150 × 70 × 50 cm) were divided into three compartments for two stimulus females (outer compartments, 45 cm) and the test male (centre compartment, 60 cm) by mesh partitions (mesh size 13 mm). The tanks were equipped with internal box filters, heaters and overhead lights, and each compartment was provided with identical hollow bricks serving as hiding place and territory focus. The fish were allowed an acclimatization period of 2 days after introduction into the experimental tank, during which males and females remained separated by the partitions but were able to communicate by visual, olfactory and acoustic signals. This part of the trial corresponds to the ‘decision phase’ of the design by Egger *et al*. (2008), during which the test fish can assess the two stimulus fish under standardized conditions. Next, males were allowed to interact freely with each of the two stimulus females at a time in ‘sequential access’ test sessions (Egger *et al*., 2008), for which the partition between the male and one of the females was removed, whereas a nontransparent panel was positioned between the male and the other female to prevent distraction of the tested individuals and manipulation of the by-standing female. In the next session, the male was given access to the other female. Access sessions lasted for 20 min after the first interaction between the pair. Intervals between access sessions were at least 1 h. Each trial consisted of three access sessions per stimulus female, and the order of access (beginning with either the homomorphic or the heteromorphic female) was alternated between trials. The scoring of unobstructed interactions between pairs had proven necessary in previous experiments, which had shown that association time and interactions through a partition did not predict mating preferences, but could represent territorial behaviour as well (Egger *et al*., 2008). Furthermore, the repetitions of the access sessions take into account that males and females interact over a longer period of time during their pre-spawning pair-bonding phase.

Males of the Moliro population (*n* = 15 males) were given a choice of one female from the Moliro population (the ‘homomorphic’ female, *n* = 19 females) and one female from one of the four other populations (the ‘heteromorphic’ female), resulting in four experiments with the four different heteromorphic populations. Heteromorphic stimulus females were from Ndole (*n* = 13 trials, five females), Chiseketi (*n* = 20 trials, eight females), a location north of Mabilibili (‘Kirschfleck’; *n* = 20 trials, eight females) and a location south of Isonga (*T. polli*; *n* = 14 trials, eight females). Logistic constraints limited the numbers of stimulus females that could be used in the experiments. Figure 1 illustrates the geographical distribution, genetic relatedness and colour patterns of the different morphs. Individuals were used in one to three trials (once, in four trials) per experiment (Table S1). Body sizes (standard length, SL) of individuals are reported as supplementary information (Table S1). Relative body size differences between males and females were calculated as RSD = (male SL − female SL)/(male SL + female SL).

Fish were observed continuously during the access sessions and components of their behaviour were scored with EthoLog 2.2 (Ottoni, 2000). Males and females court (among other behaviours) by quiver displays, and mutual courtship involves reciprocal T-positions, in which one of the pair quivers and the other one nuzzles the genital papilla of the partner (Nelissen, 1976). Courtship quivers of males and aggressive displays (charges and displays with spread fins) of males and females were tallied to provide measures of male courtship and male and female aggression respectively. Female sexual behaviour was quantified as the sum of the number of courtship quivers by the female and the number of times the female was involved in a ‘T-position’ display, that is, either presented herself to the male or let him nuzzle her genital papilla, or, with roles reversed, nuzzled the papilla of her courtship partner. Tallies were summed across the three access sessions to obtain one data point for each male-female pair.

To account for the repeated use of individual males and females, we employed generalized linear mixed models (GLMM) with male and female identities as crossed random factors. As male behaviour towards homomorphic females did not differ between experiments (see Results), data from all four experiments were combined to build models testing for morph-dependent differences in male and female behaviour. As the response variables were tallies of events, models were fitted with negative binomial error distributions (NB1 or NB2, dependent on which model achieved the higher AIC value) and log link functions, and included total observation time as offset. Analyses were conducted in R v. 2.15.0 (R Development Core Team, http://www.R-project.org) with package glmmADMB.

## Results

Behaviour of males towards homomorphic females did not differ between experiments, and there was no effect of body size asymmetry between males and females on the amounts of male aggression displays and male courtship quivers towards homomorphic females (Table S2). Courtship quivers towards homomorphic females occurred in all trials (minimum rate of 0.1 quivers per minute). In five trials (7.5% of all trials), homomorphic females received no male aggression.

**Fig. 2:**
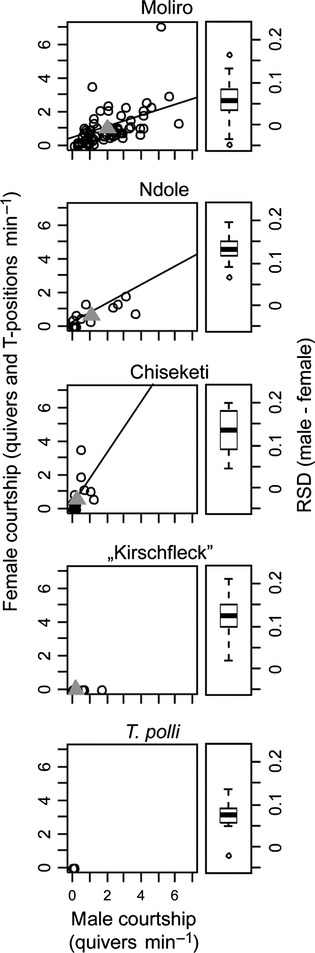
Rates of male and female courtship and body size asymmetry (RSD) between males and females. The regression lines are drawn using the intercepts and slopes estimated by the generalized linear mixed models (GLMM) reported in Table S3. Grey triangles mark the average courtship rates of males and females (omitted in the panel for *Tropheus polli*).

Homomorphic male behaviours were compared with behaviours towards females of the four different heteromorphic populations. The intensity of male courtship, scored as number of courtship quivers, towards homomorphic females was significantly stronger than towards heteromorphic females (Table 1). Moreover, the courtship intensities towards the different heteromorphic populations differed significantly from each other except for the similar quiver rates towards the Chiseketi and the ‘Kirschfleck’ females (Table 1). In particular, males courted the phenotypically and genetically similar Ndole females more intensely than both, the phenotypically similar, genetically distinct ‘Kirschfleck’ females and the phenotypically distinct, genetically related Chiseketi females. Males did not court females of the phenotypically and genetically distinct *T. polli* population (Fig. 2).

**Table 1:** Generalized linear mixed models (GLMM) estimates (intercepts and effect estimates β with standard errors SE) of the effects of female population on the rates of male courtship quivers. The model was fitted using a negative binomial error distribution (NB2) with a log link function, and male and female identity as crossed random factors. Pairwise comparisons between female populations were carried out by re-running models and alternating the population used as reference. Negative signs of the β values indicate that courtship towards the focus population was less vigorous than towards the reference population

	Reference population (intercept estimate ± SE)			
	
Focus population	Moliro (0.58 ± 0.25)	Ndole (−0.65 ± 0.41)	Chiseketi (−2.04 ± 0.36)	‘Kirschfleck’ (−2.39 ± 0.37)
Ndole	β = −1.22 ± 0.39			
	*P* = 0.0015			
Chiseketi	β = −2.62 ± 0.33	β = −1.39 ± 0.44		
	*P* = 3.8 × 10^−15^	*P* = 0.0015		
‘Kirschfleck’	β = −2.94 ± 0.33	β = −1.72 ± 0.44	β = −0.33 ± 0.39	
	*P* = < 2 × 10^−16^	*P* = 9.0 × 10^−5^	*P* = 0.40	
*Tropheus polli*	β = −7.60 ± 1.07	β = −6.38 ± 1.11	β = −4.98 ± 1.09	β = −4.65 ± 1.09
	*P* = 1.3 × 10^−12^	*P* = 9.2 × 10^−9^	*P* = 5.1 × 10^−6^	*P* = 2.1 × 10^−5^

Female courtship was observed only in encounters between the Moliro males and females from Moliro, Ndole and Chiseketi, and there was a positive correlation between male and female courtship activity (Fig. 2, Table S3). The genetically and phenotypically similar Ndole females courted Moliro males as frequently as did the homomorphic Moliro females, and the rate, at which female courtship vigour increased with increasing male quiver rates, did not differ between the two morphs. In contrast, this rate was significantly higher in females from the genetically related but phenotypically distinct Chiseketi population (Fig. 2, Table S3). Females of the genetically and phenotypically distinct *T. polli* and the genetically different but similarly coloured ‘Kirschfleck’ did not court the males at all (Fig. 2).

**Fig. 3:**
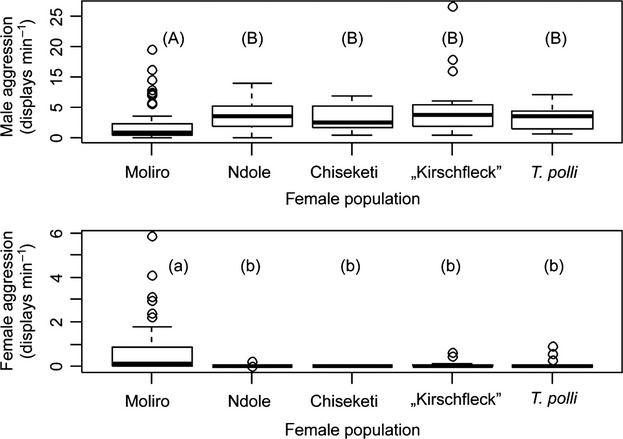
Rates of aggressive displays by Moliro males against females of different populations (upper panel), and by females of the different populations against Moliro males (lower panel). Different letters above boxplots indicate significant pairwise contrasts within panels [generalized linear mixed models (GLMM) in Tables S4 and S5].

Males directed significantly more aggressive behaviour against heteromorphic females than against females of their own population, but did not vary their levels of aggression between the different heteromorphic populations (Fig. 3; Table S4). Female aggression was displayed almost exclusively by Moliro females (Fig. 3; Table S5).

## Discussion

We found that (i) aggressive and courtship behaviour of males towards homomorphic females was independent of the morph of the alternative stimulus female, (ii) males behaved differently towards homomorphic and heteromorphic females in the contexts of both courtship and aggression, and addressed significantly more courtship and significantly less aggression to homomorphic females, and (iii) the intensity of male heteromorphic courtship, but not of aggression, depended on the females’ population, as genetically and phenotypically similar females received significantly more courtship than females of genetically and/or phenotypically distant populations. Females from genetically distant populations also showed reduced courtship activity towards Moliro males, but an opposite trend in the allocation of aggression, which they only displayed in homomorphic encounters. Aggression between males and females reflects competition for feeding territories (Yanagisawa & Nishida, 1991), and may have been determined by the interplay of body size asymmetries, which were in favour of the Moliro males especially in the heteromorphic encounters (Fig. 2), and sexual attraction between the pair, which interferes with agonistic behaviour.

Observations of different courtship intensities between different morphs are consistent with previous study on reproductive isolation in *Tropheus*, which demonstrated that discrimination against foreign populations is not universal and amongst other possible factors dependent on the similarity between the involved morphs (Salzburger *et al*., 2006; Egger *et al*., 2008, 2010, 2012). In particular, in female choice experiments with the Moliro population and another red-coloured population (Chimba; closely related to and resembling the Ndole population tested in this study), courtship was equally likely to occur within and between populations (Egger *et al*., 2010). In contrast, Moliro females exhibited strictly assortative mate choice in two-way laboratory trials and in mixed-morph breeding populations, when the alternative populations had distinct colour patterns (bluish and yellow-blotched populations), whereas Moliro males occasionally courted or mated with the heteromorphic females (Egger *et al*., 2008; Hermann, 2011; H. Brindl & K. M. Sefc, unpublished). The present data suggest that male courtship behaviour can contribute to sexual isolation between Moliro and other populations, provided that male courtship affects the probability of mating (Forsgren, 1997; Oliveira *et al*., 2000; Shamble *et al*., 2009; Lehtonen, 2012). Asymmetric courtship between Moliro males and ‘Kirschfleck’ females and between Chiseketi females and Moliro males further suggest that courtship can be displayed by one sex independent of the behaviour of the other sex. Hence, the population-dependent courtship intensities on the part of males are not necessarily reflections of female behaviour and preferences (Takahashi *et al*., 2008), but may rather result from male mating preferences and the ability of males to discriminate between populations.

The importance of male mate choice for species isolation in general is difficult to assess. The number of taxa, in which male preferences have been examined, is still rather small and the studied taxa differ distinctly in conditions known or assumed to influence the evolution of mating preferences (Parker & Partridge, 1998; Servedio, 2007), such as mating and breeding systems, patterns of current and past geographical distributions, natural and sexual selection regimes and hybrid fitness. In several taxa, only the females displayed strong assortative preferences (*Tetrix* ground hoppers, Hochkirch *et al*., 2007; darters, O'Rourke & Mendelson, 2010; guppies, Russell *et al*., 2006; *Ficedula* flycatchers, Saetre *et al*., 1997), whereas in others, males contributed significantly or exclusively to the maintenance of assortative mating (damselflies, Svensson *et al*., 2007; *Heliconius* butterflies, Jiggins *et al*., 2001, 2004; mosquito fish, Espinedo *et al*., 2010; garter snakes, Shine *et al*., 2004; cichlid fishes, Pauers *et al*., 2010; Egger *et al*., 2010; present study). Frequently, assortative male preferences were found in studies of sympatric taxa, where costs of nonassortative mating could explain the evolution of mate choice in males, for example to avoid egg predation by allomorphic females led to a stickleback male's nest (Albert & Schluter, 2004). Moreover, low hybrid fitness favours assortative mate choice in males when mating opportunities are limited (Peterson *et al*., 2005) or when courtship is associated with mortality risks, as for example due to predation on conspicuous, courting male damselflies (Svensson *et al*., 2007).

During allopatric divergence, assortative preferences can arise in both sexes for example when the social system promotes sexual imprinting and learning (Verzijden *et al*., 2012) or as a consequence of the correlated divergence of sensory systems and signals (Boughman, 2002). Indeed, assortative mate preferences of cichlids have been shown to be influenced by sexual imprinting (Verzijden & ten Cate, 2007; Verzijden *et al*., 2008) and by associations between sensory systems, signals and environmental variation (Maan & Seehausen, 2010). Both processes could theoretically be at work in *Tropheus*, as their territorial system, involving social interactions with numerous conspecifics, may be conducive to learning and individual recognition, and as divergent selection implied in colour pattern differentiation (Mattersdorfer *et al*., 2012) could also have acted on the sensory system. Mate discrimination by both sexes and the weak isolation between similar phenotypes are consistent with both sexual imprinting on and a sensory bias for homomorphic phenotypes, but discrimination between the similarly coloured but differently patterned Moliro and ‘Kirschfleck’ might be expected to be weaker if preferences were based on biased colour perception. Selection could also have targeted mate preferences directly or by reinforcement in periods when population displacements associated with lake level fluctuations resulted in secondary contact of differentiated morphs (Egger *et al*., 2007). It is certainly unlikely that each of the different pairs of morphs in this study have been interacting with each other in their past, but it has been proposed that single bouts of reinforcement may give rise to general choosiness (Coyne & Orr, 2004). Nonetheless, offspring viability and fertility in experimental crosses between some morphs (K.M. Sefc, personal observation), introgression and the potential hybrid origin of morphs (Sturmbauer *et al*., 2005; Egger *et al*., 2007), and the link between sexual isolation and genetic divergence provide no support for selection against heteromorphic mating. Furthermore, while genetically distinct lineages of *Tropheus* co-occur at some locations, it is striking that there is no sympatry of related colour variants (Konings, 1998; Egger *et al*., 2007), which would be expected if premating isolation was a product of secondary contact. Finally, a Fisherian run-away process driven by female preferences has been implied in the allopatric colour differentiation in a cichlid species complex from Lake Malawi, consistent with assortative mating in experimental situations (Knight & Turner, 2004). If mate preferences were driving colour pattern differentiation in *Tropheus*, we would expect strong discrimination already in the earliest stage of divergence, which is, however, not the case between the genetically related morphs studied here (Moliro and Ndole) and previously (Moliro and Chimba, Egger *et al*., 2010).

In previous studies of mate choice between *Tropheus* populations, the extent of colour pattern differentiation between populations covaried with their genetic distance, and it was not possible to discern whether body colour differences affected sexual isolation independently of divergence time (Egger *et al*., 2010). In this study, we compared sexual isolation in four different combinations of genetic and colour pattern differentiation, and presented Moliro males with heteromorphic choices of genetically and phenotypically similar females (Ndole), genetically and phenotypically distinct females (*T. polli*), genetically related females with distinct colour pattern (Chiseketi) and genetically distinct but similarly coloured females (‘Kirschfleck’). Males did not differentiate in their courtship rates between the latter two heteromorphic populations (Chiseketi and ‘Kirschfleck’), whereas they displayed significantly more courtship quivers to the genetically and phenotypically similar population (Ndole), and significantly less courtship towards the genetically and phenotypically distinct *T. polli*. Apparently, male discrimination was influenced not only by colour pattern differences but also by divergence in other, for example olfactory or acoustic, cues (Plenderleith *et al*., 2005; Verzijden *et al*., 2010).

Although males behaved similarly to Chiseketi and ‘Kirschfleck’ females, the females of the two populations differed in their courtship behaviour towards Moliro males. ‘Kirschfleck’ females did not respond to male courtship at all, whereas Chiseketi females showed a higher courtship rate relative to male courtship activity than for example the homomorphic Moliro females. Although the exaggerated heteromorphic courtship of Chiseketi females could be a consequence of a lack of choice offered to the females in our experiment, it is also consistent with previous observations made with females pertaining to the same yellow-blotched morph (a population from Mbita Island), which did not discriminate between homo- and heteromorphic males in laboratory two-way female choice tests against a bluish morph (Nakaku; Egger *et al*., 2010), and spawned preferentially with the bluish males in experimental ponds stocked with yellow-blotch and bluish *Tropheus* (Hermann, 2011).

Overall, mate choice data of *Tropheus* suggest that sexual isolation among populations increases with increasing genetic distance. Positive correlations between premating isolation and genetic distance have also been observed in other allopatric species pairs, including *Drosophila* flies (Coyne & Orr, 1997) and haplochromine cichlids (Stelkens *et al*., 2009). In the haplochromines, which is the cichlid clade comprising the genus *Tropheus* (Koblmüller *et al*., 2008), premating isolation was estimated to accumulate fastest during initial divergence and reach completion after 4.8/10/22 million years (Myr), depending on the underlying molecular clock (Stelkens *et al*., 2009). In our experiment, the deficiency in reproductively motivated interactions between males of the Moliro population and females of *T. polli* and the ‘Kirschfleck’ population is consistent with the proposed species delineations (Konings, 1998; Egger *et al*., 2007), provided that Moliro females do not successfully solicit matings from the foreign males, which has not been tested here. So far, however, red-morph females exerted strictly assortative mate choice in two-way and multiple choice experiments with two other distinct morphs (Egger *et al*., 2008; Hermann, 2011; H. Brindl & K. M. Sefc unpublished). Using a molecular clock calibration corresponding to the one yielding the 4.8 Myr estimate for the completion of premating isolation in the above haplochromine study, the most recent common ancestor of the *Tropheus* species complex (excluding *T. duboisi*), and hence the maximum divergence between populations, dates to approximately 1.5 Ma (Koblmüller *et al*., 2010). This does not necessarily imply that isolation evolved exceptionally fast in *Tropheus*, as premating isolation among the haplochromine species was estimated in no-choice tests and discrimination against heterospecifics in the presence of conspecific females, as in our experiments, may be achieved earlier during divergence. Accounting further for the imprecision of molecular clock dating and the different measures of premating isolation (spawning in Stelkens *et al*., 2009 vs. courtship in the *Tropheus* studies), the time span within which sexual isolation evolved among *Tropheus* populations fits well with the haplochromine model.

In conclusion, males of the Moliro population discriminate between females of different populations, and even vary their courtship behaviour between females of the same population and a phenotypically and genetically similar population. Discrimination among populations appears to increase with both, genetic distance and colour pattern dissimilarity. On the basis of current evidence, we consider it likely that assortative mate preferences evolved in both sexes as a by-product of allopatric divergence under natural or social selection. Experiments testing the effects of learning and sensory environments on mate choice are warranted to address the mechanisms underlying sexual isolation within this species complex. Understanding assortative mate preferences among *Tropheus* populations at their various stages of phylogenetic and phenotypic divergence has the potential to improve our general understanding of how allopatric populations evolve premating isolation on their way to speciation.

## Supporting information

**Table S1** Body size (standard length SL) of females and males, and the number of times each individual was used in the experiments.

**Table S2** Generalized linear mixed model estimating effects of experiment (heteromorphic female population) and RSD (relative body size difference between the male and the homomorphic female) on aggressive and courtship behaviour of Moliro males towards the homomorphic Moliro females.

**Table S3** Generalized linear mixed model estimating effects (β ± SE) of female population (Moliro, Ndole, Chiseketi) and male courtship intensity (quiver rate) on female courtship.

**Table S4** Generalized linear mixed model estimating effects (β ± SE) of female population on male aggression.

**Table S5** Generalized linear mixed model estimating effects (β ± SE) of female population on female aggression.
