# Supplementary material for: Male courtship preferences demonstrate discrimination against allopatric colour morphs in a cichlid fish
Source: J Evol Biol. 2013 Jan 3;26(3):577–86. doi: 10.1111/jeb.12074 (PMC3599476; doi:10.1111/jeb.12074)
Supplement: Supplementary file 1 [file jeb0026-0577-SD1.docx]

SUPPLEMENTARY INFORMATION

Table S1: Body size (standard length SL) of females and males, and the number of times each individual was used in the experiments.

| Individual | SL (cm) | Times used per experiment with: | | | |
| --- | --- | --- | --- | --- | --- |
|  |  | Ndole | Chiseketi | “Kirschfleck” | *T. polli* |
| Females, Moliro | | | | | |
| MWf13 | 8.8 | - | 2 | 1 | 1 |
| MWf15 | 8.8 | - | 1 | 1 | - |
| MWf16 | 9.1 | - | - | 1 | 1 |
| MWf18 | 7.8 | 1 | 2 | 1 | - |
| MWf19 | 8.6 | 1 | 2 | 2 | - |
| MWf22 | 8 | 2 | 2 | 2 | 1 |
| MWf23 | 7.5 | 2 | 2 | 1 | 1 |
| MWf29 | 9.2 | - | - | 1 | 1 |
| MWf31 | 8.6 | 1 | 1 | 1 | 1 |
| MWf32 | 8.4 | 1 | 2 | 1 | 1 |
| MWf36 | 8.4 | 2 | - | 2 | 1 |
| MWf37 | 8.8 | - | - | 1 | 1 |
| MWf38 | 8.4 | 2 | 2 | 1 | - |
| MWf40 | 8.7 | - | 1 | 1 | - |
| MWf41 | 8.8 | - | - | - | 1 |
| MWf45 | 8.8 | - | 1 | 1 | 1 |
| MWf46 | 9.5 | - | - | - | 1 |
| MWf50 | 9.7 | - | - | - | 1 |
| MWf53 | 7.9 | 1 | 2 | 2 | 1 |
| Females, Ndole | | | | | |
| Nd1 | 7.4 | 3 | - | - | - |
| Nd2 | 7 | 3 | - | - | - |
| Nd3 | 7.3 | 2 | - | - | - |
| Nd4 | 6.5 | 1 | - | - | - |
| Nd5 | 7.5 | 4 | - | - | - |
| Females, Chiseketi | | | | | |
| Ka1 | 8.4 | - | 2 | - | - |
| Ka2 | 7 | - | 2 | - | - |
| Ka3 | 7.3 | - | 3 | - | - |
| Ka4 | 7.3 | - | 3 | - | - |
| Ka5 | 6.7 | - | 3 | - | - |
| Ka6 | 7.1 | - | 3 | - | - |
| Ka7 | 6.3 | - | 2 | - | - |
| Ka8 | 6.4 | - | 2 | - | - |
| Females, “Kirschfleck” | | | | | |
| Kir1 | 7.7 | - | - | 3 | - |
| Kir2 | 7.8 | - | - | 3 | - |
| Kir3 | 6.9 | - | - | 3 | - |
| Kir4 | 7.4 | - | - | 1 | - |
| Kir5 | 7.8 | - | - | 3 | - |
| Kir6 | 6.8 | - | - | 3 | - |
| Kir7 | 6.8 | - | - | 2 | - |
| Kir8 | 7.1 | - | - | 2 | - |
| Females, *T. polli* | | | | | |
| Pol1 | 7.8 | - | - | - | 2 |
| Pol11 | 8 | - | - | - | 2 |
| Pol12 | 7.9 | - | - | - | 1 |
| Pol13 | 7.4 | - | - | - | 1 |
| Pol14 | 8.5 | - | - | - | 3 |
| Pol2 | 8.3 | - | - | - | 2 |
| Pol3 | 8.6 | - | - | - | 2 |
| Pol4 | 7.5 | - | - | - | 1 |
| Males, Moliro | | | | | |
| ML10 | 10 | 1 | - | 1 | - |
| ML11 | 10.5 | 1 | - | - | 1 |
| ML2 | 9.6 | 1 | 1 | 1 | 1 |
| MW1 | 9.3 | - | 2 | 2 | 1 |
| MW11 | 10.5 | - | - | 1 | - |
| MW3 | 9.7 | 1 | 1 | 1 | 1 |
| MW4 | 10.3 | 1 | 2 | 1 | 1 |
| MW42 | 9.5 | 1 | 2 | 1 | 1 |
| MW47 | 9.2 | 1 | 2 | 1 | 2 |
| MW48 | 9 | 1 | 2 | 2 | 1 |
| MW49 | 9.4 | 1 | 2 | 2 | 1 |
| MW6 | 9.5 | 1 | 1 | 2 | 2 |
| MW7 | 9.4 | 1 | 1 | 2 | 1 |
| MW8 | 8 | 1 | 3 | 2 | 1 |
| MW9 | 9.5 | 1 | 1 | 1 | - |

Table S2: Generalized linear mixed model estimating effects of experiment (heteromorphic female population) and RSD (relative body size difference between the male and the homomorphic female) on aggressive and courtship behaviour of Moliro males towards the homomorphic Moliro females. The model was fitted using a negative binomial error distribution (NB1) with a log link function, and male and female identity as crossed random factors. Results of likelihood ratio tests comparing models including and excluding interactions and main factors are reported.

|  | χ^2^ | Δd.f. | P |
| --- | --- | --- | --- |
| Male aggression |  |  |  |
| Experiment * RSD | 1.42 | 3 | 0.70 |
| RSD | 0.04 | 1 | 0.85 |
| Experiment | 3.40 | 3 | 0.33 |
| Male quivers |  |  |  |
| Experiment * RSD | 2.06 | 3 | 0.56 |
| RSD | 0.79 | 1 | 0.37 |
| Experiment | 3.25 | 3 | 0.35 |

Table S3. Generalized linear mixed model estimating effects (β± SE) of female population (Moliro, Ndole, Chiseketi) and male courtship intensity (quiver rate) on female courtship. Models failed to converge when experiments with “Kirschfleck” and *T. polli* females (no female courtship) were included. The model was fitted using a negative binomial error distribution (NB1) with a log link function, and male and female identity as crossed random factors. Pairwise comparisons of effect sizes between all the three morphs were achieved in two separate models, with either Moliro or Ndole as reference population (ref.).

|  | β ± SE | *P* |
| --- | --- | --- |
| Female population: |  |  |
| Ndole (ref. Moliro) | -0.70 ± 0.42 | 0.095 |
| Chiseketi (ref. Moliro) | -0.60 ± 0.31 | 0.056 |
| Chiseketi (ref. Ndole) | 0.10 ± 0.47 | 0.837 |
| Male quiver (ref. Moliro) | 0.33 ± 0.05 | **1.1*10^-11^** |
| Interactions: |  |  |
| Male quiver:Ndole (ref. Moliro) | 0.22 ± 0.17 | 0.192 |
| Male quiver:Chiseketi (ref. Moliro) | 1.19 ± 0.43 | **0.006** |
| Male quiver:Chiseketi (ref. Ndole) | 0.97 ± 0.45 | **0.032** |

Table S4. Generalized linear mixed model estimating effects (β± SE) of female population on male aggression. The model was fit using a negative binomial error distribution (NB1) with a log link function, male and female identity as crossed random factors, and “Moliro” as the reference population. Contrasts between other pairs of populations were non-significant.

| Female population (reference = Moliro) | β ± SE | *P* |
| --- | --- | --- |
| Ndole | 0.79 ± 0.31 | **0.0099** |
| Chiseketi | 0.78 ± 0.25 | **0.0016** |
| “Kirschfleck” | 0.96 ± 0.24 | **7*10^-5^** |
| *T. polli* | 0.80 ± 0.27 | **0.0031** |

Table S5. Generalized linear mixed model estimating effects (β± SE) of female population on female aggression. The model was fit using a negative binomial error distribution (NB2) with a log link function, male and female identity as crossed random factors, and “Moliro” as the reference population. Contrasts between other pairs of populations were non-significant.

| Female population (reference = Moliro) | β ± SE | *P* |
| --- | --- | --- |
| Ndole | -3.59 ± 0.84 | **1.8*10^-5^** |
| Chiseketi | -3.84 ±0.68 | **1.5*10^-8^** |
| “Kirschfleck” | -2.48 ± 0.64 | **9.9*10^-5^** |
| *T. polli* | -2.00 ± 0.74 | **0.0071** |
